# A hybrid blowfish-based cryptography and chaotic quantization steganography framework with genetic key generation

**DOI:** 10.1038/s41598-026-47129-x

**Published:** 2026-04-10

**Authors:** Rashmi Naveen, Archana Praveen Kumar, Sahana Roshan

**Affiliations:** 1 NITTE (Deemed to be University), NMAM Institute of Technology (NMAMIT), Karkala, 574110 Karnataka India; 2 Manipal Institute of Technology, Manipal Academy of Higher Education, Manipal, 576104 Karnataka India

**Keywords:** Avalanche effect, Blowfish encryption, Chaotic map, Chaotic-quant function, Genetic algorithm, PSNR, SSIM, Sustainable Development Goal, Engineering, Mathematics and computing, Physics

## Abstract

Security is the main attribute when dealing with information exchange. Confidential information theft, data loss, and data manipulation are conceivable results of security events. Different forms of data hiding are Cryptography and Steganography. Cryptography converts information into an unreadable form, and steganography hides the existence of information. The proposed work experiments with Advanced Blowfish Encryption based on an extended round function integrity with Chaotic Image Quantization (ABECIQ) as a security mechanism. ABECIQ aims to introduce a novel security mechanism that combines cryptography and steganography with the key generation scenario using a genetic algorithm. Initially, using a genetic algorithm and real-time clock values, the secret keys are created. The Blowfish algorithm’s round function ‘F’ is modified by adding crossover and mutation functions. The generated ciphertext is embedded in an image using the chaotic-quant technique. The proposed work is analysed using parameters of the Avalanche effect, Entropy values, Execution time, Attack scenario, Correlation coefficient, and Peak Signal-to-Noise Ratio (PSNR) values. The experiments demonstrate that the ABECIQ algorithm achieves PSNR values within the range of 65 to 74 dB while SSIM values are above 0.999, which indicate high imperceptibility. The generated keys also show entropy values which are close to the theoretic maximum of 8 bits per character. In addition, the proposed algorithm shows high throughput thereby indicating improved computational efficiency compared to the existing algorithm. The analysis shows that ABECIQ provides better results than the existing Chaotic, Blowfish Encryption, as well as AES-RDH algorithm. ABECIQ is evaluated with different text files of sizes 4KB and 12KB demonstrating better PSNR, MSE, SSIM, and Correlation Coefficient. In addition, the time complexity for ABECIQ has also been analyzed for embedding process.

## Introduction

Ensuring secure transmission along with storage of sensitive information is a critical challenge with the increasing use of digital media^1,2^. Data security is to protect and preserve the integrity of data^3,4^. The traditional techniques of cryptography are strong towards data protection through encryption, while steganographic methods focus on concealing the secret information within the digital media^5^. However, both approaches are not sufficient to address the evolving security threats. This is because encryption alone attracts attention while steganography alone is vulnerable to statistical attacks^6,7^.

Previous research has explored both cryptographic and steganographic methods extensively. The symmetric encryption algorithm, like Blowfish utilize substitution-permutation mechanism to encrypt data efficiently. While steganographic approaches like Least Significant Bit (LSB) embedding and chaotic-based techniques aid data hiding with various degrees of imperceptibility. The recent research has introduced hybrid methods combining both encryption and steganography to improve security. Nevertheless, each method treats key generation, its encryption, and embedding as separate component which leads to limitations in the overall randomness, computational efficiency, as well as robustness.

To address these challenges, this paper proposes a hybrid framework called ABECIQ, which combines genetic algorithm-based key generation, a modified Blowfish encryption scheme, and a chaotic quantization-based steganographic embedding technique. The integration of these components provides a multi-layered security approach, enhancing both data protection and concealment.

The primary objective of integrating steganography with cryptography is to enhance the security of sensitive information. This work aligns with the Sustainable Development Goal (SDG) 9 by advancing secure digital infrastructure through a robust cryptographic–steganographic framework^8^. ABECIQ explores novel image steganography techniques alongside advanced encryption methods. This technique has potential applications in banking, military operations, life sciences and medicine, digital watermarking, digital currency, and e-commerce. Recent research in these fields has increasingly focused on image steganography in the transform domain. By combining cryptography with steganography, an additional layer of protection is achieved. Furthermore, ABCEIQ can be extended to incorporate emerging technologies such as the Internet of Things (IoT), neural networks, and cloud computing.

The main challenge is the need to design a framework that is secure and efficient, combining key generation, encryption along with steganographic embedding, while also maintaining high imperceptibility, computational efficiency, and strong randomness. The existing techniques have often treated all these components separately, which has led to limitations in the overall performance and security of the system.

Following are the main contributions of this work

- A hybrid framework by ABECIQ that integrates generic algorithm-based key generation with a modified Blowfish encryption and chaotic steganographic embedding.
- Mechanism of enhanced key generation by utilizing a genetic algorithm, which improves both key strength and randomness.
- A modified encryption scheme which improves both confusion and diffusion properties and maintains computational efficiency.
- Ensuring high imperceptibility with PSNR values in the range of 65 to 74 dB through a chaotic quantization-based embedding method.
- A comprehensive evaluation and validation using NIST statistical tests, entropy analysis, statistical significance t-test, and RS analysis.

The remainder of the paper is structured as follows. Section “Related work” outlines the review of existing literature and methodologies. Section “Methodology of ABECIQ” presents the layout implemented in this study, detailing the experimental setup and the approaches. Section “Results and discussion” presents the empirical structure, providing details on test files and observational perspectives. Section “Evaluation” presents the perceptive hypothesis to describe the criteria used in embedding and encryption to evaluate the system’s performance. Section “Conclusion” gives the conclusion, stating suggestions for future research and highlighting the potential for further advances in information security.

## Related work

The existing related work of cryptography and steganography has advanced from traditional methods to more advanced hybrid as well as learning -based techniques. Earlier research predominantly focused on basic embedding approaches or encryption independently, while the recent work focuses on frameworks that are integrated or chaotic systems, along with parallel processing for improving efficiency and security. This section provides background on related work done in security based on the techniques used.

### Key generation and key distribution

Symmetric Key cryptography uses the same key for encryption and decryption. Distribution of this key to the sender and/or receiver requires using a trusted courier service or ending the pre-agreed key using an already secured and encrypted channel, as proposed in^9^. The possible attack on this key distribution protocol was a Man-in-the-Middle attack. To provide dual-layer security^10^, presents a strong framework for image encryption and key distribution, including sophisticated steganography, chaotic mapping, and an improved AES-128 algorithm. Encrypting dynamic and slightly altered static keys inside a QR code guarantees safe key transfer. To increase dispersion and fortify resistance against cryptographic assaults, AES includes a post-encryption shuffling procedure, a shift rows operation directed by a logistic map, and a dynamic S-box built from a two-dimensional Henon chaotic map. According to research findings, the algorithm exhibits near-perfect entropy (7.997), high UACI (~50.1%) and NPCR (>99.6%), low pixel correlation, and uniform histograms, all of which demonstrate a robust defense against noise, corruption, and divergent assaults.

Some approaches have also implemented key generation and chaotic mapping for image steganography. Lin et al. worked with Medical images^11^. The chaotic encryption algorithm based on Secure Hash Algorithm (SHA) and the adaptive Chen-based Hyperchaotic Map (CHCM) was developed to protect medical images against attacks. Another approach^12^ fused chaotic mapping and Generative Adversarial Networks (GANs) to optimize image steganography. The approach aided in balancing security and image quality. Thereby, it helped to keep the image secret with good invisibility. A robust steganography technique to protect audio was implemented on image steganography using different chaotic maps^13^. The approach integrates the Henon, Henon, and Arnold maps for encrypting images and secure audio steganography. Erkan et al. used a Convolution Neural Network (CNN) to generate the key to encrypt the image based on the chaotic logarithm map^14^. The images were encrypted using the operations of permutation, DNA encoding, diffusion, and bit reversion. These previous studies show that the key generation combined with chaotic mapping for image steganography has proved helpful and efficient, which is the novelty of the proposed research.

Another research using the Sudoku puzzle-solving mechanism^15^ provided a practical algorithm for key repository generation. This algorithm created a unique key repository from the solved Sudoku matrix. The authorized parties who had to communicate would share this key pool. The keys’ sequence would be chosen randomly from the key repository in this method during the encryption process. The index value of the first element of a key, along with the length of the key, will be sent to the recipient. One drawback of the system was its vulnerability to the solved Sudoku being captured.

Another study proposed a practical hybrid approach for bitmap-formatted image data, which utilized symmetric encryption^16^. Arman et al. used a quantum communication system and traditional hash cryptographic functions to demonstrate the possibility of continuously creating and distributing unaware keys^17^. Each technique has drawbacks due to the different methods used in the key generation algorithm.

The secret data is embedded by the Chaotic-LSB method into the LSB bits of the image pixels, wherein the embedding positions are determined by the chaotic sequence. This aids to introduce pseduo-randomness that improves resistance against statistical attacks.

### Modified block ciphers

Cryptography deals with the procedure of changing information in such a way that an eavesdropper cannot detect it citeain2025novel. Cryptography is used to jumble the information to obscure the meaning and appearance of a message. Another study tested Advanced Encryption Standard (AES), Triple Data Encryption Standard (3DES), Blowfish, and Twofish against each other in a performance comparison^18^. The study demonstrated that while Twofish is the slowest, AES provides the fastest encryption and decoding speeds, but AES takes up less memory during decryption. Finally, Blowfish and Twofish were more secure than the other algorithms while having the largest ciphertext sizes. Another study suggested that a modified blowfish technique offered higher entropy than existing cryptography methods and provided high security^19^. In the former study, keys were generated from passwords entered by users to enhance security. The former research used a single key throughout the development process for the proposed Blowfish algorithm. Using different keys for each iteration increased the robustness of the current algorithm.

Another research implemented the Blowfish method with the combination of the substitution technique^20^. Using two execution threads, parallel programming was made possible. One thread runs the substitution cipher method, while another runs the Blowfish algorithm. When comparing the proposed technique with the conventional Blowfish technique, the proposed method provides better file accuracy in the shortest amount of time. In another study, different cases for the varying round function ’F’ in the Blowfish algorithm were experimented with^21^. The other cases used to encrypt the image differed in the OR and XOR operations sequence on S-boxes. The drawback of this method was that the execution time did not vary compared to the original Blowfish algorithm, as the number of OR and XOR operations remained the same. The former research was implemented on the text files to check the security and efficiency of the mentioned cases. Each previously mentioned technique needed a complex ’F’ function that improved the security process.

Blowfish is a symmetric block cipher that is based on a Feistel network structure and operates on 64-bit data blocks. It makes use of key-dependent S-boxes and many rounds of substitution along with a permutation that provides strong diffusion and confusion properties.

### Embedding techniques

Steganography is the art of concealing information within other images, text, media, etc., to hide the secret message to make it unreadable, unlike encryption^22^. The following survey discusses how information has been concealed in images using different methods. To increase embedding capacity and imperceptibility, another study suggested combining the cross-diagonal embedding of Pixel Value Differencing (PVD) and Modulus Function (MF) approaches using edge area patterns of images^23^. Another research reviewed the Least Significant Bit Matching Revisited (LSBMR) algorithm and compared it to the Least Significant Bit (LSB) algorithm concerning the speed and compression ratio of the images^24^. Some statistical analysis methods identified the randomness of images in LSB. The hidden information was lost and could not be recovered if lossy compression was used to create the format. In another investigation, the images were only used as a point of reference while concealing data in the image’s locations^25^. The location matrix’s components were encrypted using a chaotic sequence of cover images in which the bits of the cover images were left unaltered, resulting in zero distortion. Experiments on particular color photographs yielded improved outcomes regarding data concealing capacity, i.e., in RGB bands. Matching search takes insignificant time as it has to proceed with red, green, and blue bands that might reduce the system’s performance.

### Hybrid models

One more experiment was done to improve the security of Rivest, Shamir, Adleman (RSA), where the ideas of false modulus and generalized Pell’s equation were applied^26^. Another research suggested utilizing the LSB- Blowfish and Rivest Code 4 (RC4) in combination^27^. The metrics like Mean Squared Error (MSE), Peak Signal to Noise Ratio (PSNR), and histogram analysis were used to compare the stego and the cover images to ascertain the degree of imperceptibility between them. However, LSB was a straightforward and easily detectable steganography algorithm. Hence, by offering multiple levels of security to the private data, another study highlighted using cryptography with steganography^28^. The former approach suggested encrypting and decrypting sensitive data using the AES algorithm. The generated ciphertext was embedded into the cover image using the conventional Reversible Data Hiding (RDH) algorithm. A dual security model was used to interpret the performance of LSB and RDH. For color images, the suggested algorithm produced better results. High PSNR allowed for the preservation of image quality, but little room for improvement in visual quality was observed. Enhancing the RDH process and producing more pleasing results was possible by carefully observing the visual quality.

Another study proposed the data hiding scheme of audio steganography reconciled with RC4 cryptography^29^. In the endorsed method, an audio file serves as the cover object. An audio file was subjected to a sampling procedure before the appropriate bit and cipher bit were modified. The embedding process began in the $51^{st}$ byte of the data fields, leaving the header untouched. The suggested approach was practical for audio files with higher frequency bands and offered highly secure audio. Different symmetric and asymmetric cryptography methods were experimented with using the previously mentioned method for better performance and security. Another study demonstrated how to use a variable sample selection method for audio samples by using Blowfish, one of the safest algorithms, and higher-quality audio^30^. For audio, the accomplishment of the suggested task was compared with the results of the conventional LSB approach. The proposed system was the best strategy for boosting security while maintaining the high calibre of the cover object.

Latest developments in image encryption make use of cross-image permutation and diffusion^31^. This method, through inter-image diffusion and dependency mechanism, improves image security. Similarly, to preserve important image content, parallel computing with semantically enhanced selective image encryption techniques has also used^32^. These methods work towards improving the encryption strength and their computational efficiency.

There are certain advancements in this field wherein an end-to-end CNN is used that does both image encryption and steganographic embedding to transmit the image securely^33^ . Some researchers have experimented with Lightweight Crypto-Steganography where both the techniques of cryptography and steganography are combined to secure the images^34,35^.

In contrast to the above techniques, the proposed approach combines the key generation, encryption, and steganographic embedding, thereby providing a framework which ensures both data imperceptibility and its security.

Hybrid methods that combine both cryptography and steganography aid in enhancing security. This is done by encrypting the data before it is embedded into the images, thus providing a dual-layer protection. The different techniques provided above are for encryption and data hiding. However, the methods address various aspects independently. Despite the significant progress in cryptography and steganography, existing techniques have the following limitations:

- The most significant difficulty in cryptography is key predictability, as it reduces the security of an encryption system. Weak random number generators, key production patterns, subpar algorithms, or manually selected passwords are some of the common causes of key predictability.
- A common problem in cryptography is the absence of a formal security proof, which implies that there is no mathematical assurance that a system is indeed safe. Instead of thorough formal verification, many systems are implemented based on assumptions, real-world testing, or known hard challenges.
- It alludes to the possibility that steganalysis techniques might reveal information concealed inside an image, an audio, or other types of media. This is because identification is made simpler by weak concealment techniques, obvious patterns, or subpar embedding algorithms.

In contrast, the proposed approach of ABECIQ algorithm aims to combine key generation, encryption, along with steganographic embeddding in to a single framework. By employing more robust and organized methods, the ABECIQ approach is intended to tackle typical security issues such as steganalysis vulnerability, lack of formal security evidence, and key predictability. It makes use of a sophisticated key structure and a genetic key generation algorithm. Here, the real time clock value is used as a population, so they are difficult to decipher or duplicate. By doing this, attackers are unable to guess encryption keys. The approach adheres to established mathematical concepts in the updated blowfish concept and robust methods. It frequently integrates encryption with authentication processes, enhancing dependability. Structured design facilitates the analysis and validation of security. It uses a quant-chaotic approach to conceal data. To prevent discernible patterns, changes are dispersed across the cover medium. This makes it more difficult to find or retrieve hidden data.

## Methodology of ABECIQ

To overcome the aforementioned drawbacks of the different techniques, ABECIQ uses a key generation process using a genetic algorithm that produces efficient keys. The secret keys are created using values from a real-time clock. Transmission errors are eliminated as keys are generated at the sender and receiver ends. In addition, ABECIQ also implements the Blowfish algorithm with an enhancement in the ’F’ function. The complexity of the ’F’ function is improved using the crossover and mutation process. As indicated by the high PSNR values, the proposed ABECIQ approach introduces less distortion to the cover image, thus this ensures high imperceptibility.

Furthermore, to overcome the issue of statistical analysis and reduced performance, ABECIQ calculates the low coefficients of an image to embed the data. The randomness of pixels is realized using a chaotic sequence. Moreover, the approach adds an extra layer of protection for the data by combining the technologies mentioned above, steganography, and cryptography. In addition, ABECIQ’s efficiency is measured in terms of avalanche effect, execution time, and throughput.

**Fig. 1:**
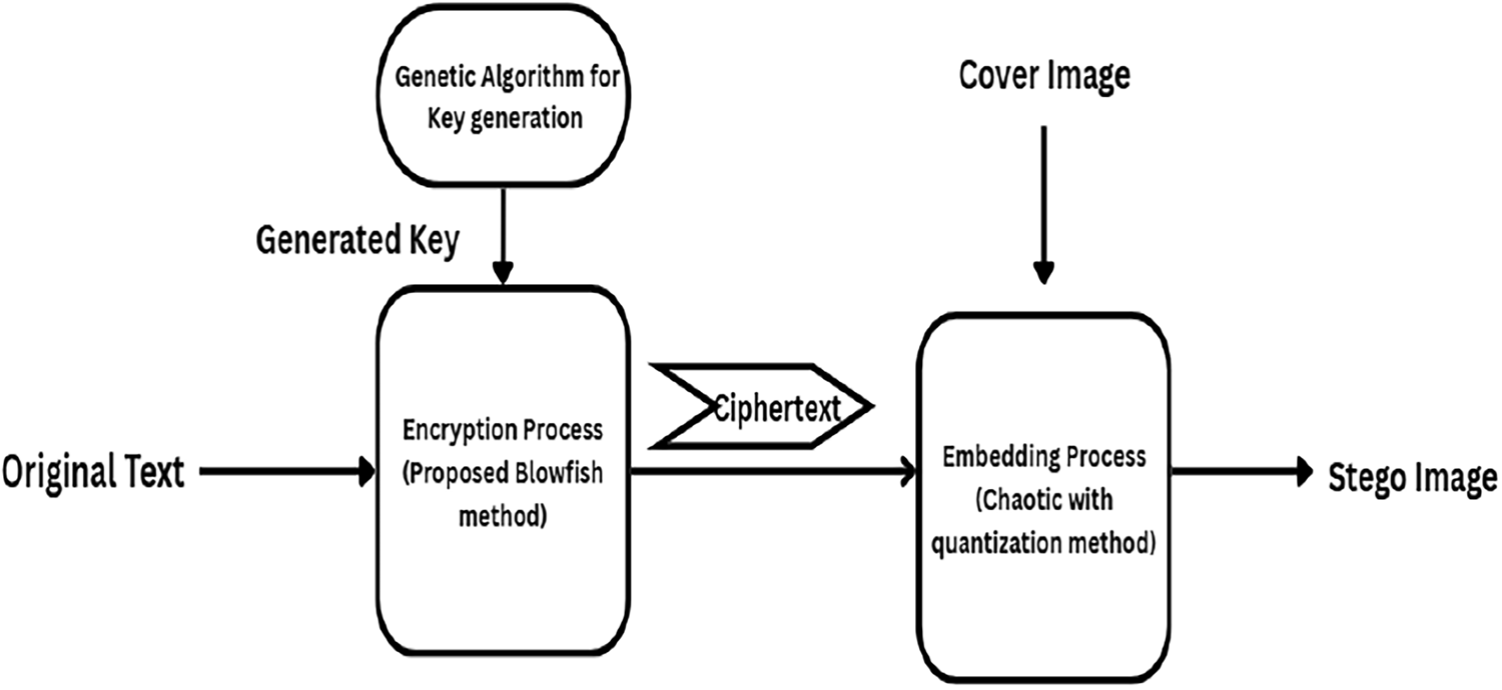
Work flow of ABECIQ crypto-stego model at the sender side.

An encryption is susceptible to attacks from a variety of angles. However, data encryption is crucial for most security plans because it can prevent an attacker from accessing private information. It is preferable not to consider encryption to be the only security measure. Therefore, ABECIQ illustrates the use of combined cryptography and steganography techniques for better performance and security. The basic layout of the suggested model at the sender side, called the Encoding-Embedding Process, is illustrated in Fig. 1.

The inverse operation is applied on the receiver side to get the initial text back. At the receiver end, the backtrack method extracts the stego image to get the ciphertext and decrypts the ciphertext to get the original text. This inverse operation at the receiver side is called the Extraction-Decoding process. The Encoding-Embedding Process is explained in the “Encoding-embedding process”, while the Extraction-Decoding Process is briefed in the “Extraction-decoding process”.

### Encoding-embedding process

The sender is responsible for the encoding and embedding procedure. And the final product obtained is the stego image. The suggested approach encrypts and decrypts data using an enhanced version of the Blowfish algorithm. For the embedding process, the chaotic method with quantization is implemented. The ABECIQ model on the sender side is implemented by executing the following steps:

1. Obtain the efficient key for the symmetric cryptography from the genetic key generation algorithm.
2. Perform symmetric encryption with the updated Blowfish technique on the original text.
3. Embed the obtained ciphertext into a cover image using image steganography with a chaotic quantization method.

All three steps above are explained in the following subsections of the “Creation and distribution of keys”, “Encryption–updated blowfish scheme”, and “Embedding–chaotic method with quantization”.

#### Creation and distribution of keys

**Fig. 2:**
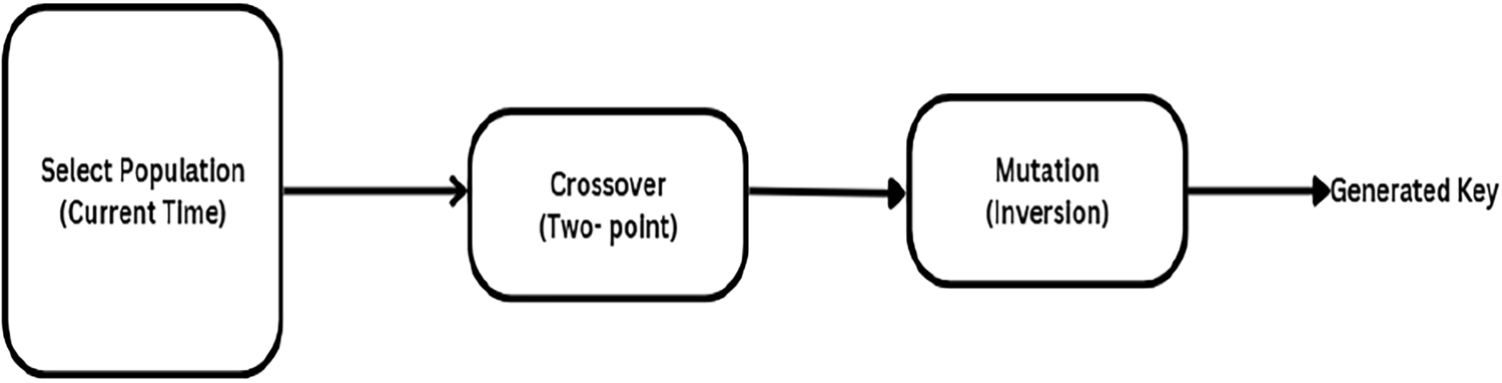
Steps in genetic algorithm.

The keys used in Blowfish encryption differ in length from 32 bits to 448 bits. Among the research, a study^36^ mentioned the technique of creating keys using a genetic algorithm and a real-time clock. The former research proved to be efficient in terms of entropy values. Hence, ABECIQ examines the performance criteria concerning the entropy values. Figure 2 shows the steps used in the genetic algorithm.

The key is generated as a result of the above steps. Key generation and distribution are the main obstructions in symmetric cryptography. A genetic algorithm with a real-time clock is used to overcome the problem as shown in Algorithm 1.

#### Encryption–updated blowfish scheme

The symmetric encryption technique is best suited for vast amounts of data. The suggested Blowfish method uses the key produced in the first step as input for the encryption process. With a modification in the round function, ABECIQ employs the Blowfish encryption method in this step. The Blowfish is one of the most accepted symmetric cryptography methods in which encryption and decryption use the same key. Therefore, the input key is known only to the source node and considered the destination node, as mentioned in^37^.

**Fig. 3:**
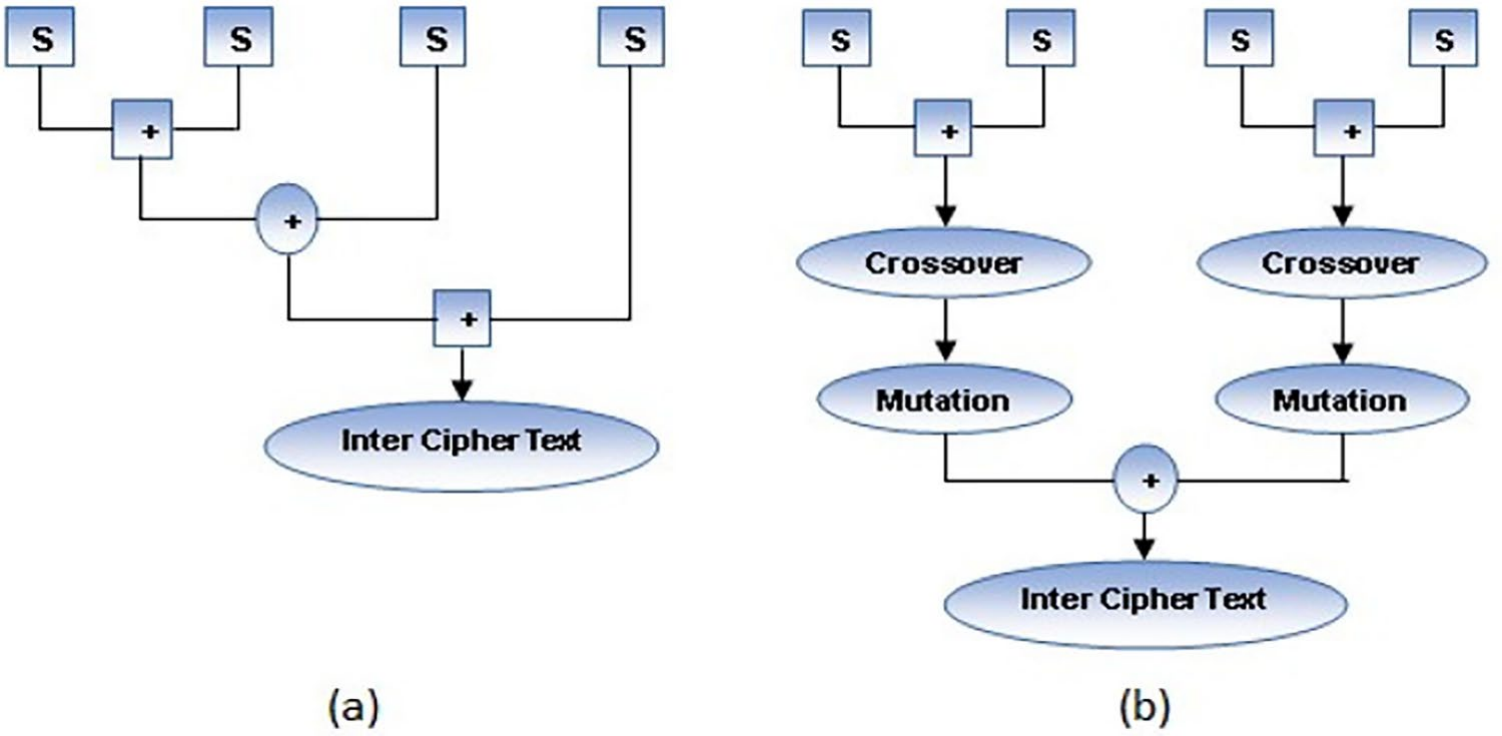
(**a**) Round function in existing Blowfish (**b**) Round function in ABECIQ.

The Blowfish algorithm requires complicated calculations to carry out rounds for various key sizes. Plain text in the Blowfish undergoes 16 rounds before pre-processing. Each round accepts a different key as input. Each round function key is obtained by performing an XOR of a unique random key for a specific round and the generated key. A schematic of the single-round function of the Blowfish technique is illustrated in Fig. 3a. Each round function uses 8 S-boxes (Substitution boxes), which process 8 bits each. The sequence of OR, EX-OR, and OR operations is executed on these S-boxes to get an intermediate ciphertext. Among the quickest encryption algorithms (block ciphers) is Blowfish. The ABECIQ approach combines crossover and mutation techniques separately on pairs of S-boxes to create a complicated intermediate ciphertext that makes it harder for an attacker to alter the final ciphertext. The first step in the genetic algorithm is Crossover in which two chromosomes are merged to form a new chromosome. It is divided into three types - one point, two point and uniform crossover. Crossover points are selected randomly. The proposed system uses two-point crossover, in which two crossover points are selected randomly. After performing crossover, mutation is performed on the resultant chromosome. In the final mutation process, new offspring are obtained by complementing the resultant chromosome obtained as a result of the crossover function. The following steps are executed to generate the Inter cipher text as shown in 1. The workflow of a single round function with proposed work for 4 S-boxes is depicted in Fig. 3b.

**Algorithm 1:**
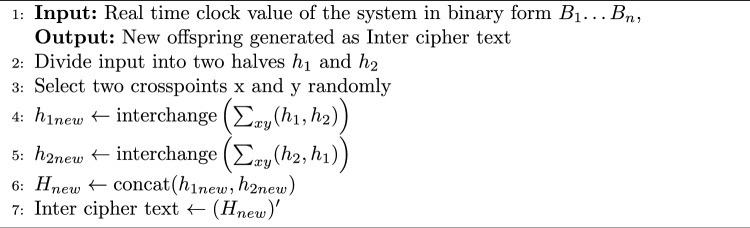
Genetic Algorithm in Key Generation Process

The ABECIQ algorithm for encryption is discussed in Algorithm 2. The Algorithm 2 explains how the Encryption process is carried out for a given plaintext P of 256 bits. The selected population size is 50–100. If the population size is too small, it may lead to a failure mode where a stable point can be found very early, which might not be the optimal solution. With the increasing population size, the computational power increases without respective advantages^38^. These aspects lead to a moderate population size, balancing computational power and convergence. With crossover rate = 0.8, most individuals participate in recombination, accelerating convergence. With a mutation rate= 0.02, only a small number of genes mutate, preserving solution quality while maintaining diversity. High crossover accelerates improvement, while low mutation preserves stability and avoids random search behavior. Large-scale search space exploration is encouraged by using a relatively high crossover rate. Low mutation rate preserves the dependability of a superior solution. The decryption process is the reverse of encryption, with the reverse sequence of keys. Table 1 shows the analysis of Crossover & mutation rates for different population sizes.

The Algorithm 2 combines the genetic algorithm operations within a block cipher framework, thereby enhancing randomness and security. The input plaintext is divided into eight segments, each of 32 bits, that are processed in pairs, thereby generating the intermediate values using XOR operations. These values then undergo two-point crossover and a probabilistic bit-flip mutation, which introduces diffusion and nonlinearity. The intermediate blocks resulting from this are then combined to form the candidate ciphertext that is again refined with round-specific subkeys through the logical operations. The candidate solutions are evaluated using the fitness function that is based on nonlinearity, entropy, and avalanche effect, thereby ensuring the identification and selection of the intermediate ciphertext, which is very secure. This process is applied iteratively across multiple rounds to obtain the final ciphertext.

**Table 1:** Analysis of crossover rate and mutation rate.

Population size (with respect to 256 bits)	Crossover rate	Mutation rate	Diversity	Behavior
Small Population: 2–6 bits	0.6–0.79	0.025–0.05	Low	Probability of early stagnation
Medium Population: 10–20 bits	0.72–0.89	0.008–0.02	Medium	Optimal balance
Large Population: 40–100 bits	0.81–0.93	0.002–0.01	High	Reliable but delayed
Very large population: >200 bits	0.83–0.95	0.0007–0.004	Very High	High overhead & complexity

**Algorithm 2:**
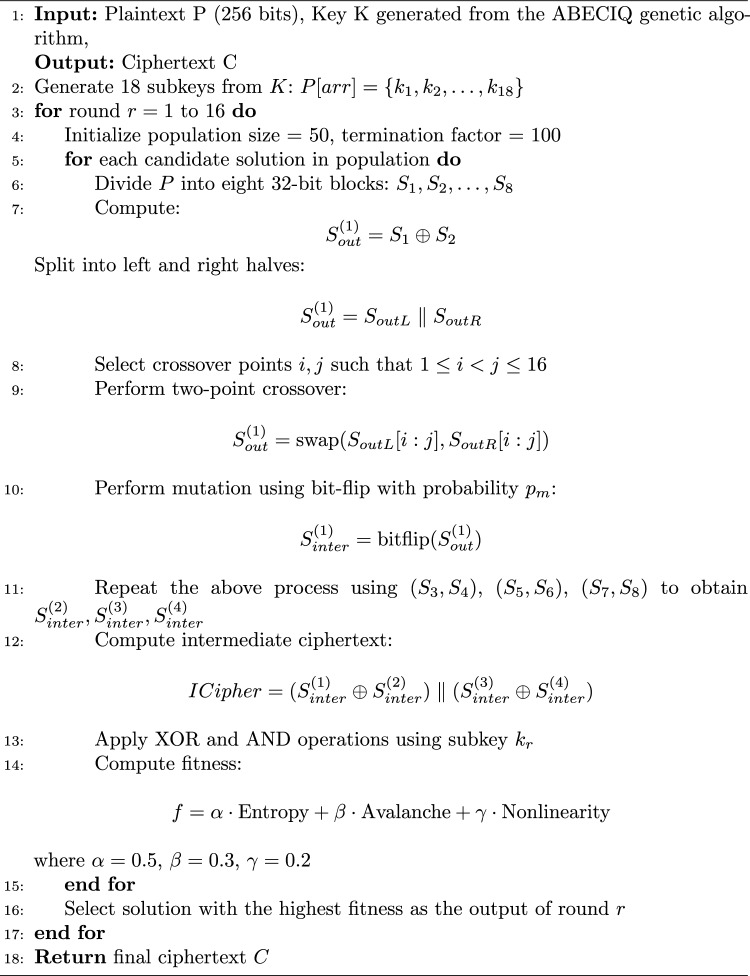
Algorithm for Encryption

#### Embedding–chaotic method with quantization

The main goal of a steganographic technique is its data hiding capacity and attack resistance capacity. Steganography is an ancient technology, and many steganography algorithms are available today. Different performance characteristics are considered during testing, including the ability to conceal data, the complexity of an algorithm, the domain being used, and the degree of attack resistance.

A chaotic matrix is used to create and store a chaotic sequence. For the extraction procedure, the receiver receives this chaotic matrix. The following formula in Eq. (1) is used to calculate the chaotic sequence. For a given current pixel value X_n_ and $\mu$=3.6, the new pixel value X_n+1_ is derived using Eq. (1).

1 $$\begin{aligned} X_{n+1} = \mu X_n (1 - X_n) \end{aligned}$$

where $X_n \in (0,1)$ denotes the current value of the chaotic sequence, $X_{n+1}$ is the subsequent value, and $\mu$ is the control parameter governing the system dynamics. For values of $\mu$ typically in the range $3.57 < \mu \le 4$, the sequence exhibits chaotic behavior, which is utilized to generate pseudo-random embedding positions.

**Fig. 4:**
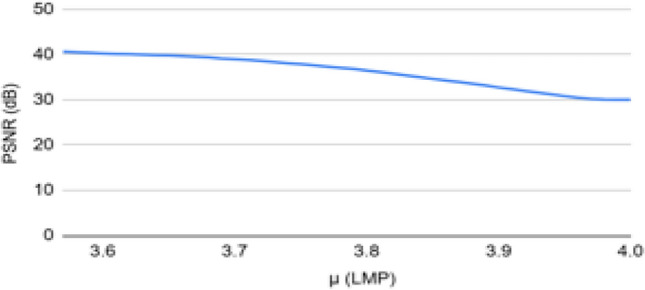
PSNR vs $$\mu$$.

**Fig. 5:**
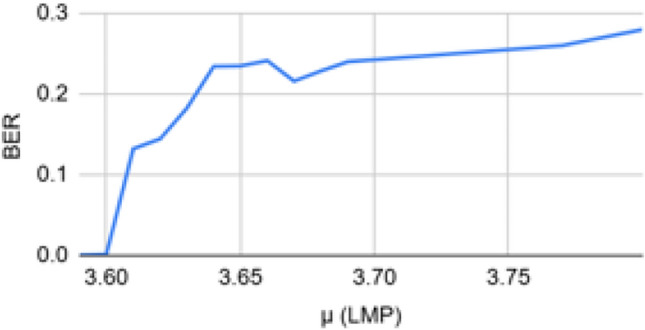
BER vs $$\mu$$.

The study in^25^ experimented with the values of $\mu$ for the chaotic sequence. If the value of $\mu$ is between 0 and 1, the output pixel value is 0. If it’s between 1 and 3, the system achieves equilibrium. And if it’s more than 1+$\sqrt{6}$, the system exhibits chaotic behaviour and oscillates between infinite values. The values between 3.57 and 4 show proper chaotic behaviour. Hence, in the ABECIQ system, to create a chaotic sequence, the value of each pixel is calculated by considering the value of $\mu$ = 3.6. A single bit variation in the initial condition can alter a chaotic sequence significantly. Even though the system is deterministic, this occurs due to the system’s high sensitivity to the initial conditions. The value of $\mu$=3.6 has been chosen by comparing the values of PSNR and Bit Error Rate (BER) with $\mu$. BER is the proportion of bits received incorrectly to the total number of bits transmitted^39^. For a range of $\mu$ values from 3.57 to 4, it is observed that PSNR is at its peak and comparatively constant about $\mu$= 3.6 and decreases as $\mu$ rises approaching 4.0, whereas Bit Error Rate (BER) increases after $\mu$= 3.6 value. The same can be observed in the Figs. 4 and 5; hence, the optimal value of 3.6 is chosen for $\mu$.

As discussed in^40^, the former technique has the following shortcomings. Large payloads decrease the effectiveness of Portable Network Graphics (PNG) compression, frequently resulting in a compressed image larger than the original. This method is also incompatible with lossy compression formats like Joint Photographic Expert Group (JPEG), and the stego image is easily detectable even without the original image. With the introduction of the chaotic map, the security of LSB steganography is improved, but the image quality cannot be improved.

ABECIQ emphasizes using a chaotic algorithm with the quantization technique, which overcomes the problem of the chaotic method with the LSB algorithm. For the generated chaotic sequence, the proposed system uses a quantization method. Quantization transforms continuous, infinite values into discrete, finite values that are easier to handle. A small coefficient has a very subtle effect on the actual data. Even if minor weights were removed from an image, the image would still be very similar. In the ABECIQ, the chaotic sequence with small coefficients is replaced with hidden data bits. Equation (2) is used to compute the coefficients’ coeff’ for an m $\times$ n image.

2 $$\begin{aligned} coeff&=(C_{i})(C_{j})(total) \end{aligned}$$

3 $$\begin{aligned} C(i)&= {\left\{ \begin{array}{ll} \frac{1}{\sqrt{m}}, & i = 0 \\ \sqrt{\frac{2}{m}}, & 1 \le i \le m-1 \end{array}\right. } \end{aligned}$$

4 $$\begin{aligned} \quad C(j)&= {\left\{ \begin{array}{ll} \frac{1}{\sqrt{n}}, & j = 0 \\ \sqrt{\frac{2}{n}}, & 1 \le j \le n-1 \end{array}\right. } \end{aligned}$$

5 $$\begin{aligned} \textit{total}&= \sum _{k=0}^{m} \sum _{l=0}^{n} \text {pixel}[k][l] \left( \frac{\cos ((2k+1)k\pi )}{2m} \right) \left( \frac{\cos ((2l+1)l\pi )}{2n} \right) \end{aligned}$$

where Eqs. (3), (4) and (5) represent the values of $C_{i}$, $C_{j}$ and total.

The ABECIQ algorithm for embedding is presented in Algorithm 3. Uniform locus selection with two-point crossover is executed, where each locus is selected from either parent with probability 0.5. The mutation operator separately flips each bit with a likelihood to operate on a single^41^. The formal definition of the mutation operator is given in Eq. (6), and Eq. 7 shows the required cases of the mutation operator.

6 $$\begin{aligned} a'= & \mathcal {M}(a) = (a_1', a_2', \ldots , a_n') \end{aligned}$$

7 $$\begin{aligned} x_i'= & {\left\{ \begin{array}{ll} 1 - x_i, & \text {with probability } p_m \\ x_i, & \text {with probability } 1 - p_m \end{array}\right. } \quad \forall i \in \{1, 2, \ldots , n\} \end{aligned}$$

In image steganography, mutation in key generation improves security by increasing unpredictability and entropy in the generated key or embedding pattern.^42^

**Algorithm 3:**
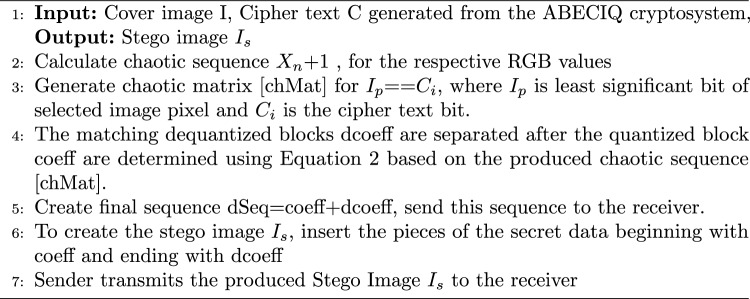
Algorithm for Embedding

### Extraction-decoding process

The ABECIQ system executes the reverse encoding-embedding model at the receiver side. The stego image is the input, and the original text is the output of the ABECIQ system. The basic steps of the ABECIQ extraction-decoding process are as follows.

1. The receiver receives the stego image from the sender.
2. The extraction process is carried out with the reverse stego algorithm.
3. The generated ciphertext, as a result of step 2, is fed as input into the decryption process.
4. The mentioned decryption algorithm (reverse of the encryption algorithm in 2 is executed and generates the expected original text.

## Results and discussion

This section discusses the significant accomplishments of this work. The ABECIQ has been compared to existing algorithms based on the criteria used in the former experiments. Hence, in this experiment, different existing works have been compared with the ABECIQ algorithm for each metric. The ABECIQ key generation algorithm provides the highest security with the generated keys compared to the existing key generation algorithm. ABECIQ does not allow an attacker to predict the plaintext simply through statistical analysis. The system’s throughput is better than that of the existing one. According to the accuracy results, the decrypted file contains no extra noise. The ABECIQ quant-chaotic technique enhances the stego image’s quality. The main intentions of information security are fulfilled with the ABECIQ crypto-stego model. A general description of the files analyzed and the results obtained by experimenting with the suggested system is provided in this part. All the above-mentioned observations have been explained in the following Sections.

### Test files

The ABECIQ algorithm is tested to ensure conformity. Python 3.9.6 is used for the implementation of the ABECIQ crypto-stego model. Using an Intel®CoreTM i11 1135G7 processor 4.2 GHz machine, a benchmark to calculate execution time describes how well a program performs. Encryption experiments are conducted on text files ranging in size from 10 KB to 47 KB. Different images from 6 KB to 83 KB are analyzed for steganography, and text files of various sizes are embedded in these images. The images are taken from the Signal and Image Processing Institute database of the University of Southern California.

### Observation perspectives

This subsection provides the detailed execution scenario of a) Generated keys, b) Execution time required for the ABECIQ algorithm, and c) Histograms of original images and stego images from the ABECIQ embedding technique in the following subsections.

#### Generated keys

**Fig. 6:**
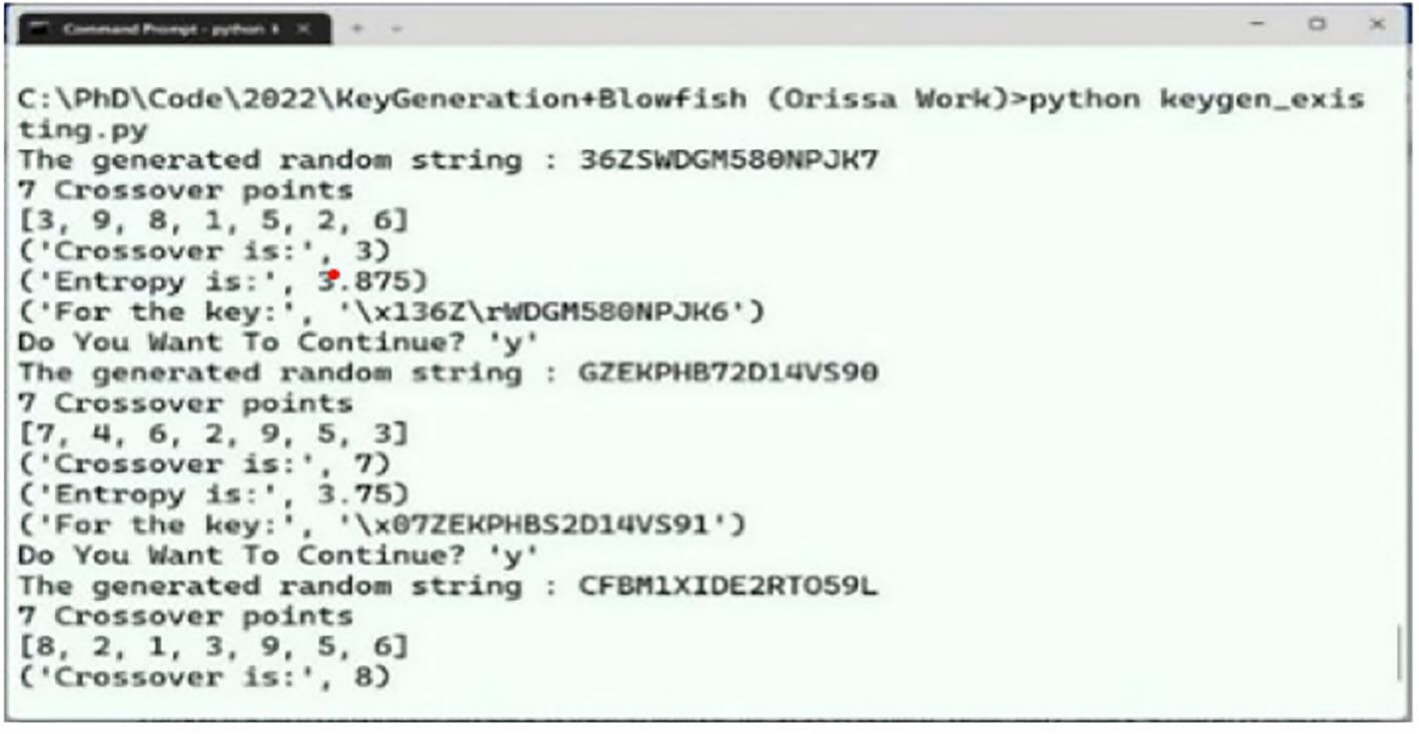
Execution scenario of generated keys using existing method.

**Fig. 7:**
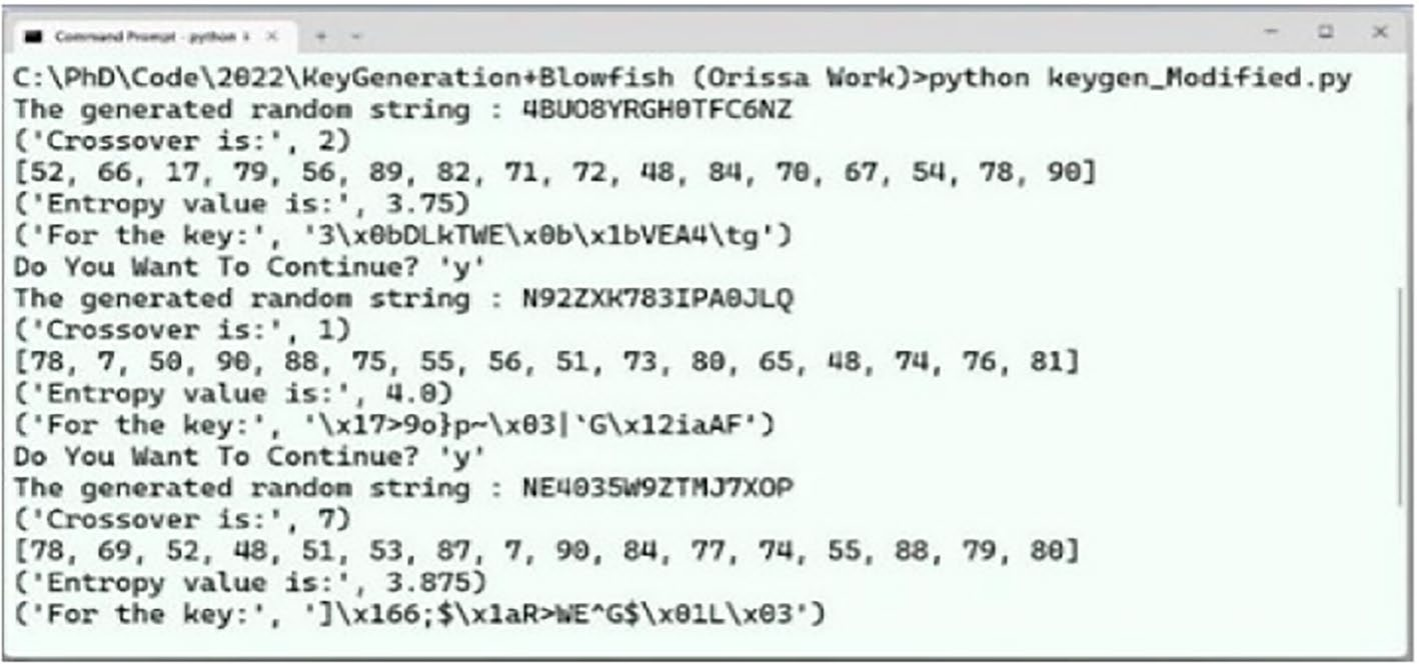
Execution scenario of generated keys using ABECIQ.

Both the existing and ABECIQ key generation algorithms are used to produce keys. Figures 6 and 7 illustrate the execution scenario of the keys generated using the existing and ABECIQ methods. $K_{e}$ represents the key generated by the existing key generation algorithm, while $K_{\text {gen}}$ represents the key generated by the ABECIQ algorithm. The values of 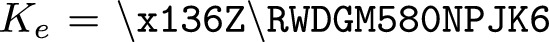, and the value of 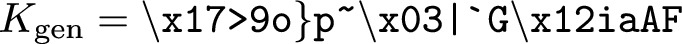. It can be observed that the ABECIQ technique generates a key with a high entropy (bits/char) of approximately 8. The existing Blowfish algorithm generates the key with an entropy (bits/char) value of 7.75.

#### Encryption

**Fig. 8:**
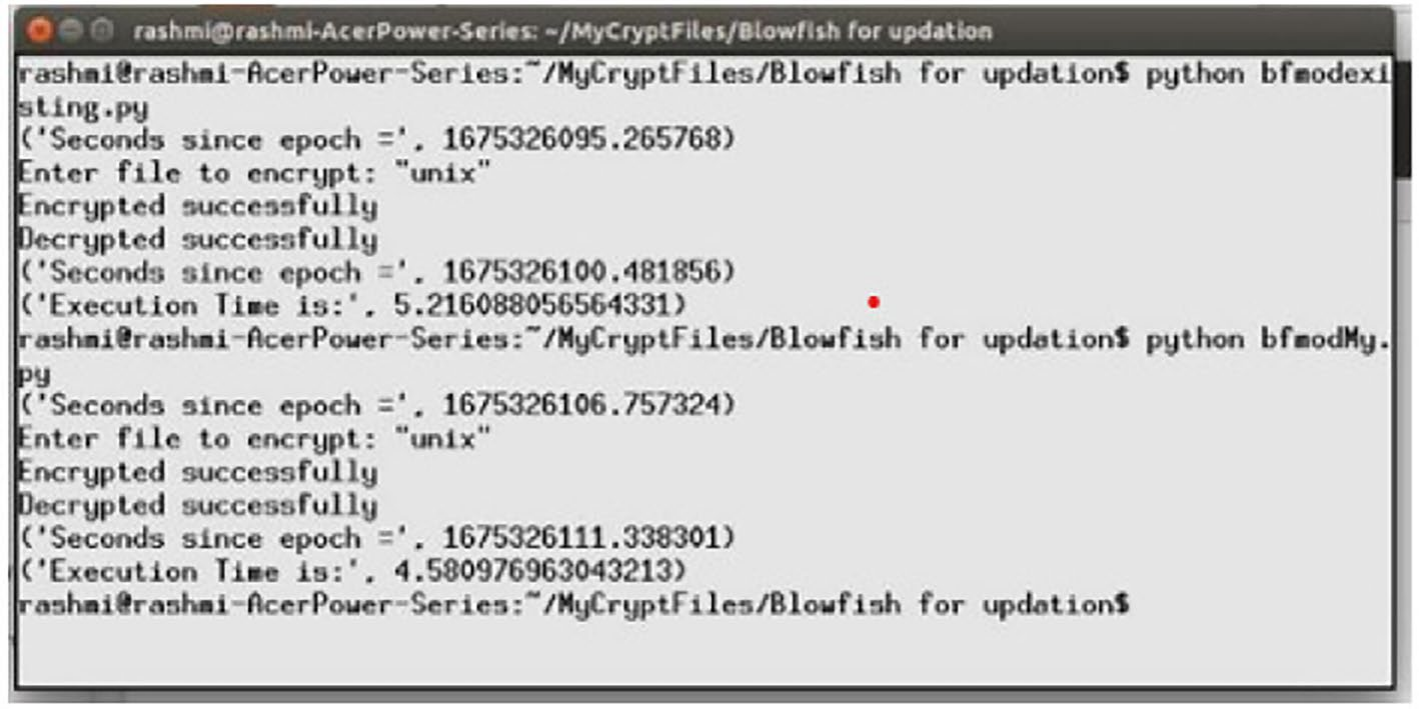
Execution time scenario for ABECIQ.

The ABECIQ method is applied to text files ranging in size from 10 KB to 47 KB. Figure 8 depicts the screenshot of the execution time required for the existing and ABECIQ algorithms for the file unix.txt (47KB). As observed in the Fig. 8, it can be seen that the existing algorithm has an execution time of 5.216 seconds compared to that of the ABECIQ algorithm, which is 4.5809 seconds.

#### Embedding

**Fig. 9:**
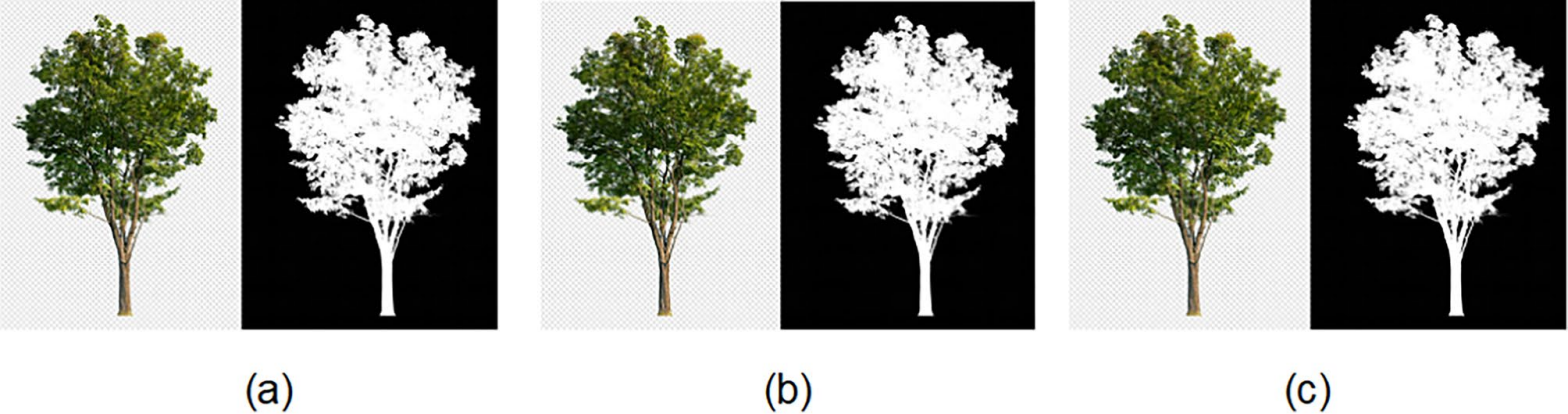
Embedding results: (**a**) Original tree image (**b**) After embedding 4KB text (**c**) After embedding 12KB text.

**Fig. 10:**
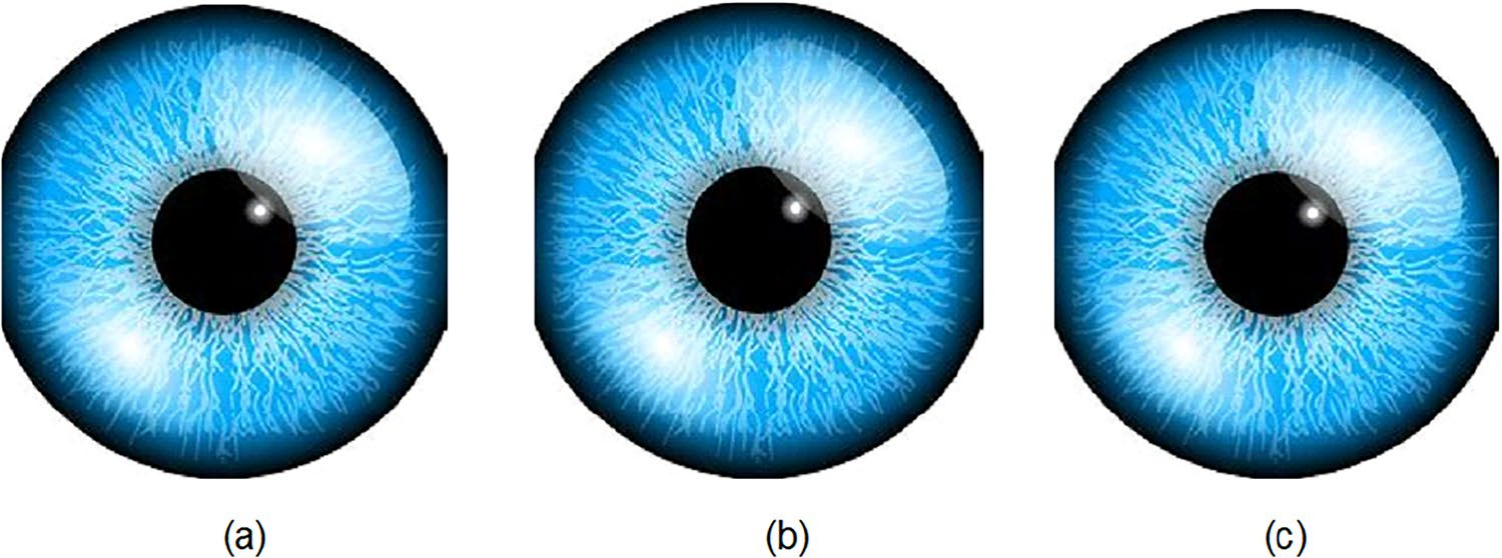
Embedding results: (**a**) Original eye image (**b**) After embedding 4KB text (**c**) After embedding 12KB text.

Quant-Chaoo embedding method is executed on images from 6 KB to 83 KB for varying text files. The embedding results before and after embedding is shown in Figs. 9 and 10. The original data is accurately reconstructed in the decryption; however, as a visual image output is not produced, it is not shown in figure form.

**Fig. 11:**
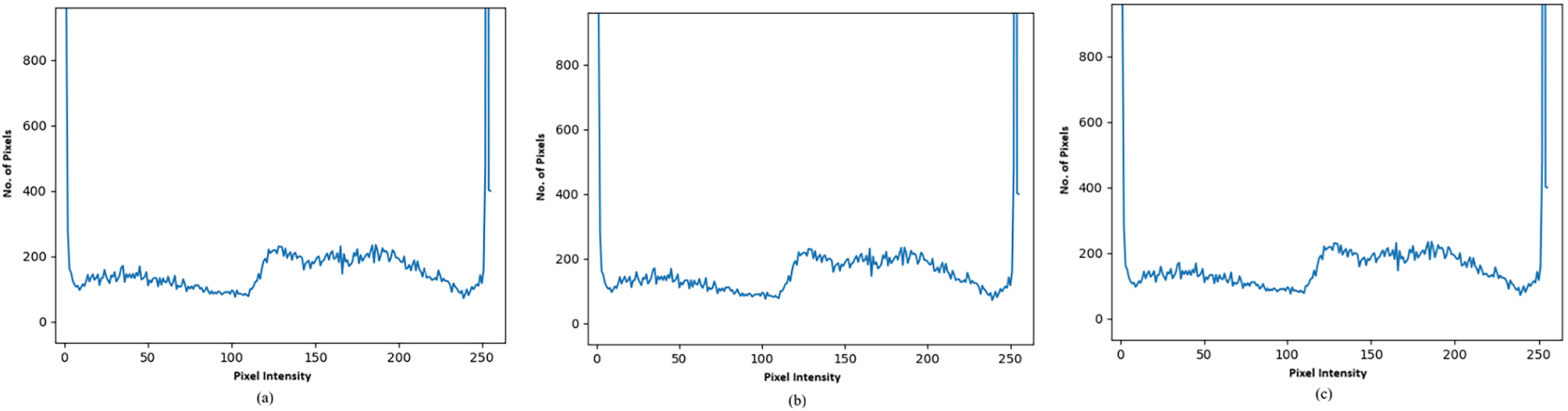
Histogram of test results utilized in the investigation (**a**) eye.png (83 kb) (**b**) eye.png stego file after embedding 4 kb text file (**c**) eye.png stego file after embedding 12 kb text file.

Figure 11a shows the histograms of the original image eye.png, and Fig. 11b shows the histogram of eye.png after embedding 4 KB of data.

**Fig. 12:**
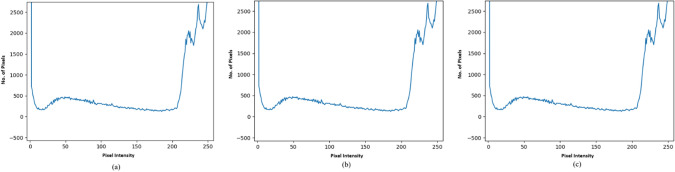
Histogram of test results utilized in the investigation (**a**) tree.png (51 kb) (**b**) tree.png stego file after embedding 4 kb text file (**c**) tree.png stego file after embedding 12 kb text file.

In comparison, Fig. 11c shows the histogram of eye.png after embedding 12 KB of data. Similarly, Fig. 12a show the histograms of the original images tree.png, while Fig. 12b,c show their relevant stego images after embedding 4KB and 12KB data, respectively.

## Evaluation

The following subsections describe the encryption and embedding criteria used to evaluate the extent to which the ABECIQ algorithm performs concerning the generated keys, encryption, and steganography.

### Key generation

The security of generated keys is calculated using entropy values and an attack scenario. The ABECIQ method gives better random values for the key. The brute-force attack scenario is analyzed. The results show that the ABECIQ algorithm takes a long time to break the key for online and offline attacks.

#### Entropy value

In both academic and practical settings, the strength of cryptographic keys is a persistent problem. Entropy is a metric used to assess how random a data-generating function is. The complete entropy data has no discernible patterns because it is uneven. Future values can be predicted using data that has low entropy. Equation (8) represents the formula for calculating entropy. N represents the total number of observed occurrences in the equation, and $P_{i}$ is the probability of the $i^{th}$ event.

8 $$\begin{aligned} H = - \sum _{i=1}^{n} p_i \log _2 p_i \end{aligned}$$

where *H* represents entropy, $p_i$ denotes the probability of occurrence of the *i*-th symbol, and *n* is the total number of symbols.

The strength and effectiveness of cryptographic functions are evaluated using entropy. The keys produced by the modified and current methods are shown in Table 2. The table compares the performance of the ABECIQ algorithm with the existing algorithm in terms of the generated key values and their entropy values. It can be observed that the length of the $K_{gen}$ is comparatively more extended than that of $K_{e}$ with stronger entropy values.

**Table 2:**
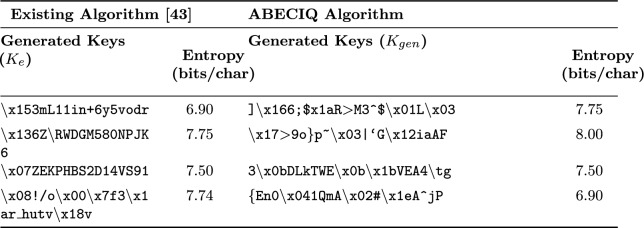
Entropy values for the generated keys.

9 $$\begin{aligned} H_{\text {normalized}} = \frac{H}{8} \end{aligned}$$

where $H_{\text {normalized}}$ represents the normalized entropy, *H* is the computed Shannon entropy of the generated key, and the value 8 denotes the maximum possible entropy (in bits per character) for an 8-bit representation.

The entropy is computed using Shanon Entropy formula 8 over the distribution of characters in the generated key. As each character is denoted using 8 bits i.e., ASCII encoding the maximum possible entropy is also 8 bits per character. Hence, the reported entropy values are represented in bits per character. The entropy is normalized by dividing the entropy per bit by 8. These entropy values do not represent the total entropy of the key i.e., 128 bits, but the average randomness present per character. Table 3 shows the entropy values expressed using Shannon entropy expressed in bits/character. Normalized entropy is obtained by using Eq. (9). The average entropy obtained for the given keys is 0.9421.

**Table 3:**
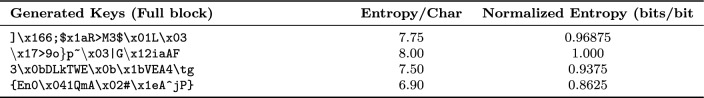
Entropy analysis of generated keys.

#### Security of keys

A brute force search analysis was performed using an external estimation tool so that an intuitive understanding of attack feasibility could be obtained. However, such tools are not technically designed for rigorous evaluation of the cryptographic algorithm; the proposed method’s security is appropriately characterized by its key space. For 128 bits of key size, the possible number of keys existing is $2^{128}$, which is computationally infeasible. This is because there will be a need for an exhaustive search over $2^{128}$ keys even if an attacker tests $10^{12}$ per second. Hence, the proposed system achieves a security level that is comparable to the standard cryptographic systems. Table 4 shows the Security analysis based on key space and attack complexity. From the table, it can be observed that since the existing approach does not state the fixed key length, there can be no direct comparison in terms of brute-force. Hence, the comparison is done only in terms of search-space and key representation. According to the analysis, it can be observed that the ABECIQ key generation algorithm takes longer to search the selected password and has a vast search space of $2^{128}$ for online and offline attack scenarios.

**Table 4:** Security analysis based on key space and attack complexity.

Measures or Algorithm used	Existing Algorithm^43^	ABECIQ Algorithm
Key representation	GA–TPM based key parameters	Fixed-length binary key
Key size specification	Not explicitly defined in bits	128 bits
Search space / key space	Parameter-dependent (not expressed as $2^n$)	$2^{128}$
Brute-force complexity	Depends on GA/TPM parameters	$O(2^{128})$
Online attack scenarios	Limited by system constraints and authentication controls	High resistance due to large key space
Offline attack scenarios	Parameter-dependent exhaustive search	Computationally infeasible under standard assumptions

Note: The method compared here does not provide a fixed key size as the genetic algorithm, and Tree Parity Machine parameters derive the key. Hence, the comparison is only provided in terms of computational complexity and key space characteristics rather than the time-based estimations

### NIST—statistical validation of generated keys

The randomness and cryptographic strength of the generated keys are validated using the NIST Statistical Test Suite i.e., NIST SP 800-22. This validates and evaluates the randomness properties of the sequences using multiple statistical tests, such as runs, entropy-based, and frequency tests. The keys generated are subjected to these tests, and their corresponding p-values are analyzed to determine whether there is true randomness among the sequences. Table 5 shows the NIST Statistical Test for the obtained criteria. The generated sequences are evaluated using the NIST SP 800-22 statistical test suite at a significance level of 0.01. All fifteen tests satisfied both the P-value and proportion criteria. The results indicate statistical randomness.

**Table 5:** NIST statistical test results for generated keys.

S.No.	Test	P-value	Proportion	Required ($\ge$96)	Result
1	Frequency (Monobit)	0.9421	99/100	Yes	Pass
2	Block Frequency	0.8874	98/100	Yes	Pass
3	Runs Test	0.9012	97/100	Yes	Pass
4	Longest Run of Ones	0.8735	98/100	Yes	Pass
5	Binary Matrix Rank	0.9123	99/100	Yes	Pass
6	Discrete Fourier Transform	0.8642	97/100	Yes	Pass
7	Non-overlapping Template	0.9311	98/100	Yes	Pass
8	Overlapping Template	0.8895	97/100	Yes	Pass
9	Maurer’s Universal	0.9188	99/100	Yes	Pass
10	Linear Complexity	0.9057	98/100	Yes	Pass
11	Serial Test	0.8946	97/100	Yes	Pass
12	Approximate Entropy	0.8763	98/100	Yes	Pass
13	Cumulative Sums	0.9215	99/100	Yes	Pass
14	Random Excursions	0.8882	97/100	Yes	Pass
15	Random Excursions Variant	0.9034	98/100	Yes	Pass

### Encryption

The suggested method independently uses crossover and mutation techniques on pairs of S-boxes to produce a complicated intermediate ciphertext and improve resistance against tampering. The following subsections provide a detailed analysis of the ABECIQ algorithm concerning execution time, throughput, and throughput effect.

#### Avalanche effect

A distinctive tendency of encryption algorithms where a slight change in input, like flipping a single bit, results in a vast and unforeseen change in the output is known as the “avalanche effect” in cryptography^44^. Any encryption algorithm would find the avalanche effect as one of its most prudent characteristics. A slight alteration in the plaintext or the key should significantly alter the ciphertext. The term “avalanche effect” refers to this characteristic. It measures how the slight modification to the plain text or key impacts the ciphertext. In other words, if one bit in the entered string is changed, the hash value’s bit count should change by at least 50%. A good encryption algorithm should always fulfil the relation- Aval eff>50% as discussed in^45^.

An encryption algorithm may indulge in a quick statistical analysis if it doesn’t meet this requirement. This means that, if changing just one bit of the input causes only one bit of the desired output to change, it is simple to decrypt the encrypted text. Avalanche effect is calculated using Eq. (10). In the equation, cbits is the number of changed bits in the ciphertext, while cipherbits are the bits in the ciphertext as a whole.

10 $$\begin{aligned} {\text {Aval eff}}=\frac{{\text {cbits}}}{{\text {cipherbits}}} \end{aligned}$$

**Fig. 13:**
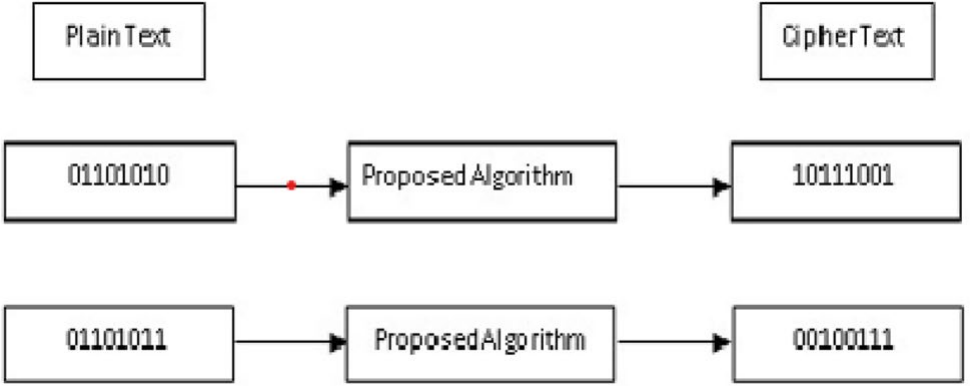
Analysis of avalanche effect.

Table 6 shows the average avalanche effect for experimental text files, and Fig. 13 shows the sample analysis of 8-bit text for avalanche effect, where a one-bit alteration to the plaintext results in the ciphertext changing by five bits. The same can be observed from Table 6 that the ABECIQ algorithm is better regarding the Avalanche impact than the existing algorithm.

**Table 6:** Comparison of avalanche impact (in percentage).

Modification to the plain text’s bit count	Existing Blowfish Encryption^21^	ABECIQ
1 bit	57.89	58.55
2 bits	57.89	57.89
3 bits	55.92	57.23
4 bits	50.01	52.63

Strict Avalanche Criterion states that changing 1 bit of input should change half of the output bits^46^. The Strict Avalanche Criterion requires that complementing a single input bit results in each output bit changing with probability 0.5. Experimental evaluation of the proposed scheme yields an average Strict Avalanche Criterion value of 0.4882, which closely approximates the ideal value and confirms strong diffusion characteristics. For 256 inputs and 8 bit flips, 2048 tests are performed. Average bit change is calculated using the following Eq. (11).

11 $$\begin{aligned} \text {Average bits changed}=\frac{\text {Total changed bits}}{\text {Total tests}} \end{aligned}$$

If the total bit changes are 9986 then Average bits changed= 9986/2048=4.875. Table 7 shows the Strict Avalanche Criteria for ABECIQ algorithm with average value 0.4882.

**Table 7:** Average Bit Change for S-Boxes.

**S-Box**	**Average Bit Change**
1	4.892
2	4.875
3	4.870
4	4.891

#### Differential cryptanalysis

In differential cryptanalysis, $\Delta X$ represents the difference between two plaintexts that an attacker deliberately selects to study how differences propagate through the encryption algorithm. The Eq. (12) shows the differential cryptanalysis formula.

12 $$\begin{aligned} \Delta X = P_1 \oplus P_2 \end{aligned}$$

where:

- $$P_1$$ = first plaintext
- $$P_2$$ = second plaintext
- $$\oplus$$ = XOR operation
- $\Delta X$ = input difference (chosen difference pattern)

Differential cryptanalysis, introduced by Eli Biham and Adi Shamir^47^, evaluates the propagation of input differences through nonlinear components of a cipher. If certain input differences consistently produce predictable output differences, the cipher may be vulnerable. The security of the proposed scheme is primarily governed by the differential behavior of its 4 S-boxes. In the presented algorithm, an encryption process is carried out for a given plaintext P of length 256 bits. Here, we try to intentionally select two plaintexts with a specific bit pattern difference to observe how that difference affects the ciphertext, which helps to identify weak S-box behaviour.

For input difference, $\Delta X = 0x3F$, Eq. (13) gives the output difference $\Delta Y$.

13 $$\begin{aligned} \Delta Y = S(x) \oplus S(x') \end{aligned}$$

where *x* is the original text and $S(\cdot )$ represents the S-box.

For the above $\Delta X$, the obtained output difference is $\Delta Y = 0x6D$. This procedure is repeated 256 times to calculate the Differential Distribution. Table 8 shows the few cases of the number of occurrences for the respective output differences.

**Table 8:** Differential cryptanalysis results.

Output differences ($\Delta Y$)	No. of occurrences
0x37	3
0x6D	4
0x5F	2
0x2B	1
Others	0 to 3

Here, the largest count is 4. The probability for the 256 possible outputs is calculated using Eq. (14)

14 $$\begin{aligned} P = \frac{4}{256} = 2^{-6} \end{aligned}$$

This means that for any chosen input difference, the most frequently occurring output difference appears only 4 times out of 256 possibilities, giving a probability of $2_{-6}$ which matches the optimal bound for strong S-boxes.

#### Execution time

The total time required to perform the encryption and decryption is called the execution time. The time required to execute the existing Blowfish and ABECIQ algorithms is recorded. Table 9 contains the speed benchmarks (in seconds) for the existing and ABECIQ algorithms. Table 9 shows the execution time in seconds for the different text files, comparing the ABECIQ and the existing algorithm^21^. For each file, it can be easily seen that the ABECIQ algorithm executes faster than the existing algorithm.

**Table 9:** Comparison of Execution time in seconds.

Text files	Existing blowfish encryption^21^	ABECIQ
Try.txt (10KB)	2.23	2.10
Lab2.txt (15KB)	3.75	3.35
Lab1.txt (26KB)	5.04	4.01
Unix.txt (47KB)	5.21	4.58

The time complexity for the proposed Blowfish algorithm is as follows: For the key generation, it is O(1), and for the encryption (total blocks P) it is O(P). Table 10 shows the mean and standard deviation for the multiple runs. The combined statistical analysis indicates that ABECIQ achieves lower average execution time with minimal variance compared to Blowfish, demonstrating both efficiency and stability across multiple runs.

**Table 10:** Comparison of execution time in seconds for multiple runs.

Files	Run	Existing blowfish encryption^21^	ABECIQ
Try.txt (10KB)	1	2.23	2.10
	2	2.16	2.02
	3	2.21	2.08
	4	2.23	2.07
	Avg	2.23	2.10
Lab2.txt (15KB)	1	3.75	3.35
	2	3.80	3.39
	3	3.70	3.32
	4	3.65	3.26
	Avg	3.75	3.35
Lab1.txt (26KB)	1	4.95	3.94
	2	5.04	4.01
	3	4.99	3.96
	4	5.08	4.04
	Avg	5.04	4.01
Unix.txt (47KB)	1	5.21	4.58
	2	5.17	4.52
	3	5.25	4.60
	4	5.10	4.46
	Avg	5.21	4.58

The execution time analysis for different text files is performed over multiple runs, and the results are reported as mean ± standard deviation to reflect the variability and reliability of the measurements. The result is shown in the Table 11.

**Table 11:** Mean and standard deviation.

File (size)	Blowfish mean	Blowfish SD	ABECIQ mean	ABECIQ SD
Try.txt (10KB)	2.23	0.029	2.09	0.026
Lab2.txt (15KB)	3.75	0.053	3.35	0.046
Lab1.txt (26KB)	5.04	0.059	4.01	0.045
Unix.txt (47KB)	5.21	0.041	4.58	0.042

#### Throughput

Throughput of the encryption scheme is calculated as shown in Eq. (15). Here $b_{i}$ represents the total number of bits in plaintext, and E shows the Encryption time.

15 $$\begin{aligned} T=\frac{\sum b_{i}}{E} \end{aligned}$$

Table 12 shows the difference between the data processed (in bits/sec) with the existing algorithm^21^ and the ABECIQ algorithm. It can be observed that the ABECIQ algorithm achieves higher throughput than the existing algorithm across the different text files. This indicates an improved computational efficiency. However, it is important to note that throughput is a performance metric, and this metric does not directly indicate cryptographic security.

It can be observed that the ABECIQ algorithm achieves higher throughput than the existing algorithm across different text files, indicating improved computational efficiency. However, throughput is a performance metric and does not directly reflect cryptographic security.

**Table 12:** Throughput comparison.

Text files	Existing blowfish encryption^21^	ABECIQ
Try.txt (10KB)	0.3424	0.4504
Lab2.txt (15KB)	0.4576	0.5970
Lab1.txt (26KB)	0.6787	0.7481
Unix.txt (47KB)	1.3157	1.4336

### Statistical analysis

To validate and to check if the observed improvements are statistically significant, a t-test is conducted between the results of the proposed approach and the existing algorithm across multiple test cases. Below is the statistical significance analysis using a two-sample (independent) t-test for 5 runs per file. The sample sizes are $n_1 = n_2 = 5$, with a significance level $\alpha$ = 0.05, Degrees of freedom $df = 8$. The Critical t-value for two-tailed test at $\alpha$ = 0.05, $df=8$ is approximately 2.306. For this assumption table shows the t-test. As shown in Table 13 all computed t-values exceed the critical values, thereby proving that the differences between the proposed ABECIQ algorithm and the existing Blowfish algorithm are statistically significant. The result of a statistical t-test is considered to be statistically significant when the computed t-value is greater than the critical value of 2.306.

**Table 13:** Statistical analysis using t-test.

File	Mean (Blowfish)	Mean (ABECIQ)	t-value	Significance
Try.txt (10KB)	2.20	2.05	7.91	Significant
Lab2.txt (15KB)	3.65	3.25	12.78	Significant
Lab1.txt (26KB)	5.14	4.11	31.37	Significant
Unix.txt (47KB)	5.19	4.56	23.75	Significant

### Image steganography

Effectiveness, the difference between two pictures, and the imperceptibility of the embedding process are some of the primary criteria frequently used to assess the success of image steganography techniques. This effectiveness can be evaluated based on PSNR, MSE, Structural similarity index (SSIM), and Correlation coefficient^48^. In this section, all the aforementioned metrics are used to calculate the performance of the ABECIQ algorithm. The proposed approach is also compared with the reversible data hiding methods based on common image quality metrics.

#### PSNR and MSE

An accurate comparison of the amount of compression noise introduced into the signal, which impacts how accurately it is represented, is designated by the Peak signal to noise ratio (PSNR) measure^20^. By contrasting the traits of the original image and the stego image, the stego image quality is assessed. It determines the measurable error. The resulting stego image in the above work is slightly altered because the cover image conceals the ciphertext. Stego picture quality is evaluated using two metrics: MSE and PSNR.

The average squared difference between the original image’s and the stego image’s pixel values is measured by M E. Let I be the matrix of the original ima e. Let K be the matrix of stego images. The picture’s dimensions are m $\times$ n (height $\times$ width). Equation (16) represents the formula for M E. The PSNR value in quantization for n-bits is expressed in Eq. (17).

16 $$\begin{aligned} \text {MSE}= & \frac{1}{MN} \sum _{i=1}^{M} \sum _{j=1}^{N} \left[ I(i,j) - K(i,j) \right] ^2 \end{aligned}$$

17 $$\begin{aligned} \text {PSNR}= & 10 \log _{10} \left( \frac{MAX^2}{\text {MSE}} \right) \end{aligned}$$

where MSE is the mean squared error and MAX is the maximum possible pixel value of the image.

The PSNR values are verified using the standard formula as per the equation, based on the MSE values computed, thereby ensuring consistency across the results. The results show a high PSNR value, which is due to the minimal modifications in the pixel that are introduced during the embedding process. Since ABECIQ alters only a small portion of the LSB the distortion is extremely low, resulting in high PSNR and low MSE value.

All PSNR values have been verified using the standard formula based on the computed MSE values, ensuring numerical consistency across the results.

#### SSIM

The SSIM is a technique for predicting how well the viewers will receive digital television and film images and other kinds of digital images and videos^48^. SSIM is used to measure similarity between two images. Structural information suggests that the neighbouring pixels are closely associated and thus affect one another. This is particularly true when considering spatial interactions, especially those between nearby pixels. Local regions of two images are compared, and the SSIM index for pixel x and y is calculated as shown in Eq. (18). Here $\mu$x, $\mu$y are the sample means of x and y, while $\delta$x and $\delta$y are the variances of x and y. $C_{1}$ and $C_{2}$ are to stabilize the division.

18 $$\begin{aligned} SSIM(x,y)=\frac{(2\mu x\mu y+C_{1})(2\delta x\delta y+C_{2})}{(\mu x^{2}+\mu y^{2}+C_{1})(\delta x^{2}+\delta y^{2}+C_{2})} \end{aligned}$$

The suggested strategy and the current technique’s PSNR, MSE, and SSIM metrics are displayed in Tables 14 and 15. SNR is included as an additional metric to evaluate the signal quality of the stego image.The ABECIQ algorithm is compared with the existing algorithm concerning PSNR, MES, and SSIM in the two Tables 14 and 15. Tables 14 compares the PSNR, SSIM, and MSE for text size of 4 kb with^25^, while Table 15 compares the parameters for text size of 12 kb with^25^. The ABECIQ algorithm has also been compared with another algorithm with^28^ in Table 16 for a text size of 4kb to give an idea of the full potential of the proposed algorithm. The Table 16 gives performance comparison on Standard Image Quality Metrics. It can be observed that the existing algorithm has less PSNR, more MSE, and less SSIM compared to the ABECIQ algorithm for most image files of different sizes with the two existing algorithms. Here, AES-RDH is a reversible data hiding technique, and it is compared with the proposed approach, which is non-reversible. Hence, the comparison is limited only to the quality assessment without any direct methodological equivalence.

**Table 14:** Performance comparison of existing chaotic-LSB and ABECIQ algorithm for text size 4 kb - PSNR (in db), SSIM (between − 1 and 1) and MSE.

	Existing Chaotic-LSB Algorithm^25^				ABECIQ Algorithm			
Image files	PSNR	MSE	SSIM	SNR	PSNR	MSE	SSIM	SNR
Eye.png (83 KB)	61.82	0.06862	0.99971	55.77dB	66.53	0.02167	0.99995	64.77dB
Global.png (13.6 KB)	61.90	0.06734	0.99959	59.85dB	66.76	0.01989	0.99991	65.15dB
Rose.png (6.75 KB)	61.25	0.07727	0.99925	59.24 dB	65.27	0.02913	0.99973	63.49 dB
Sun.png (15.5 KB)	69.96	0.00997	0.99950	68.15 dB	70.53	0.00217	0.99997	74.76 dB
Tree.png (51.3 KB)	67.46	0.01663	0.99996	65.92 dB	71.86	0.00434	0.99998	71.75dB
Monalisa.png (25 KB)	62.89	0.01015	0.99961	68.06 dB	64.32	0.00283	0.99971	73.62 dB
Tulip.png (30 KB)	63.18	0.01016	0.99963	68.05 dB	74.32	0.00203	0.99971	75.05 dB
Taiwan.png (32 KB)	65.34	0.01023	0.99964	68.03 dB	69.51	0.00302	0.99987	73.33 dB

**Table 15:** Performance comparison of existing chaotic-LSB and ABECIQ algorithm for text size 12 kb - PSNR (in dB), SSIM (between − 1 and 1).

	Existing Chaotic-LSB Algorithm^25^			BECIQ Algorthim		
Image files	PSNR	MSE	SSIM	PSNR	MSE	SSIM
Eye.png (83 KB)	56.58	0.00041	0.99998	59.78	0.09910	0.99942
Global.png (13.6 KB)	48.02	0.20037	0.99924	60.2	0.08942	0.99977
Rose.png (6.75 KB)	Size not compatible					
Sun.png (15.5 KB)	65.47	0.03014	0.99840	85.14	0.00050	0.99998
Tree.png (51.3 KB)	62.54	0.05364	0.99988	66.05	0.01771	0.99997
Monalisa.png (25 KB)	58.45	0.00120	0.99987	59.15	0.08702	0.99989
Tulip.png (30 KB)	60.54	0.06219	0.99988	66.05	0.01771	0.99997
Taiwan.png (32 KB)	60.86	0.07291	0.99989	64.26	0.03528	0.99991

**Table 16:** Performance comparison on standard image quality metrics (for text size 4 kb - PSNR (in dB), SSIM (between -1 and 1) and MSE).

	Existing AES-RDH Algorithm^28^			ABECIQ Algorthim		
Image files	PSNR	MSE	SSIM	PSNR	MSE	SSIM
Eye.png (83 KB)	50.77	0.00089	0.99946	66.53	0.02167	0.99995
Global.png (13.6 KB)	51.16	0.00086	0.99930	66.76	0.01989	0.99991
Rose.png (6.75 KB)	47.30	0.2004	0.99920	65.27	0.02913	0.99973
Sun.png (15.5 KB)	51.48	0.00085	0.99937	70.53	0.00217	0.99997
Tree.png (51.3 KB)	65.5	0.03022	0.99860	71.86	0.00434	0.99998
Monalisa.png (25 KB)	61.87	0.06829	0.99959	64.32	0.00283	0.99971
Tulip.png (30 KB)	72.99	0.07083	0.99968	74.32	0.00203	0.99971
Taiwan.png (32 KB)	63.11	0.00574	0.99959	69.51	0.00302	0.99987

Note: AES-RDH, a reversible data hiding technique, while the proposed approach is non-reversible. Therefore, the comparison is limited to image quality metrics and does not imply direct methodological equivalence

#### Mean and standard deviation

To evaluate the variability across multiple runs, the mean and standard deviation of PSNR, MSE, and SSIM values are computed as follows. The mean ($\mu$) represents the central or average value of a dataset. The following Eq. (19) the mean of PSNR, MSE, and SSIM values.

19 $$\begin{aligned} \mu = \frac{1}{N} \sum _{i=1}^{N} x_i \end{aligned}$$

where, $\mu$ = mean; *N* = number of observations (here 30 runs); $x_i$ = independent values.

Standard deviation ($\delta$) measures how much the values vary from the mean. The following Eq. (20) is used to calculate the standard deviation.

20 $$\begin{aligned} \sigma = \sqrt{\frac{1}{N-1} \sum _{i=1}^{N} (x_i - \mu )^2} \end{aligned}$$

where $\sigma$ represents the standard deviation, *N* is the total number of observations, $x_i$ denotes the *i*-th observed value, and $\mu$ is the mean of the observations. A chaotic initial seed is the starting value used to generate a chaotic sequence in a chaotic system. Since chaotic systems are extremely sensitive to initial conditions, even a tiny change in the initial seed produces a completely different sequence. The experimental results in Table 17 represent the mean ± standard deviation over 30 independent runs with different chaotic initial seeds. The proposed ABECIQ algorithm demonstrates improved imperceptibility and stability compared to the existing Chaotic-LSB method.

**Table 17:** For text size 4 kb – mean & standard deviation Analysis.

Image	Algorithm	PSNR (dB)	MSE	SSIM
Eye.png	Existing Chaotic-LSB	$61.82 \pm 0.48$	$0.06862 \pm 0.0031$	$0.99971 \pm 0.00005$
Eye.png	ABECIQ	$66.53 \pm 0.29$	$0.02167 \pm 0.0012$	$0.99995 \pm 0.00002$
Global.png	Existing Chaotic-LSB	$61.90 \pm 0.44$	$0.06734 \pm 0.0029$	$0.99959 \pm 0.00006$
Global.png	ABECIQ	$66.76 \pm 0.31$	$0.01989 \pm 0.0010$	$0.99991 \pm 0.00003$
Rose.png	Existing Chaotic-LSB	$61.25 \pm 0.52$	$0.07727 \pm 0.0038$	$0.99925 \pm 0.00008$
Rose.png	ABECIQ	$65.27 \pm 0.33$	$0.02913 \pm 0.0016$	$0.99973 \pm 0.00004$
Sun.png	Existing Chaotic-LSB	$69.96 \pm 0.21$	$0.00997 \pm 0.0006$	$0.99950 \pm 0.00004$
Sun.png	ABECIQ	$70.53 \pm 0.18$	$0.00217 \pm 0.0002$	$0.99997 \pm 0.00001$

#### Correlation coefficient

The correlation coefficient r is used in image steganography analysis to analyze the relationship between adjacent pixels, particularly to ascertain how embedding impacts image structure or to find hidden data. Perfect positive correlation (r=1) means that the values of the two pixels rise in proportion to each other. This suggests a high degree of structural similarity^49^. No correlation (r=0)–there is no linear relationship between the statistically unrelated pixel values. Perfect negative correlation (r=-1) means that when one pixel value rises, the other falls proportionally. In natural photographs, this is uncommon. By calculating the linear correlation between the respective pixel intensities, Pearson’s correlation coefficient (r) is frequently used to measure how comparable an original and stego image are. With $x_{m}$ representing the $m^{th}$ pixel in the first image and $y_{m}$ representing the $m^{th}$ pixel in the second, Eq. (21) provides the method to perform Pearson’s test.

21 $$\begin{aligned} r = \frac{\sum (x - \mu _x)(y - \mu _y)}{\sqrt{\sum (x - \mu _x)^2 \sum (y - \mu _y)^2}} \end{aligned}$$

where *x* and *y* represent pixel values of adjacent pixels, and $\mu _x$ and $\mu _y$ denote their mean values.

Table 18 shows both the stego and original images’ correlation coefficients. Tests are conducted for the suggested and present approaches for different font sizes. It can be observed from Table 18 that the correlation coefficient for the ABECIQ algorithm is much better than that of the existing algorithm. This shows that the ABECIQ algorithm performs better regarding the correlation coefficient for the embedded text files.

**Table 18:** Correlation coefficient.

	Embedded text files 4KB		Embedded text files 12KB	
Image files	Existing Chaotic Algorithm	ABECIQ Algorithm	Existing Chaotic Algorithm	ABECIQ Algorithm
Eye.png (83 KB)	0.999995	0.999998	0.99998	0.99999
Global.png (13.6 KB)	0.999994	0.999998	0.999981	0.999994
Sun.png (15.5 KB)	0.999997	0.999999	0.99997	1
Tree.png (51.3 KB)	0.9999993	0.9999997	0.999997	0.999999
Monalisa.png (25 KB)	0.999997	0.999998	0.999980	0.999990
Tulip.png (30 KB)	0.999998	0.999998	0.999981	0.999991
Taiwan.png (32 KB)	0.999998	0.999999	0.999985	0.999992

Table 19 shows the mean ± standard deviation across the seven images for the two payload sizes. The proposed ABECIQ algorithm shows slightly lower standard deviation and higher correlation than the existing chaotic algorithm, especially at 12 kb payload size images. These results show the improved embedding and robustness of the proposed approach.

**Table 19:** Statistical evaluation of correlation coefficients (mean ± standard deviation) across 7 images for two payload sizes.

Payload size	Method	Mean ± std. dev
4 KB	Existing Chaotic Algorithm	0.999996 ± 0.000002
4 KB	Updated Chaotic Algorithm	0.999998 ± 0.000001
12 KB	Existing Chaotic Algorithm	0.999982 ± 0.000008
12 KB	Updated Chaotic Algorithm	0.999994 ± 0.000004

#### RS analysis

RS analysis is a statistical steganalysis technique used to detect LSB-based steganography in images. It evaluates regular (R) and singular (S) pixel groups to estimate the presence and size of hidden payloads. To perform RS analysis, group pixels into non-overlapping blocks, define a flipping function F, and compute a discriminating function for each group as follows.

22 $$\begin{aligned} f(G)= & \sum _{i=1}^{n-1} \left| x_{i+1} - x_i \right| \end{aligned}$$

23 $$\begin{aligned} f(F(G))= & \sum _{i=1}^{n-1} \left| F(x_{i+1}) - F(x_i) \right| \end{aligned}$$

The above Eqs. (22) and (22) the regular, singular or unchanged group depending on the following conditions. If f(F(G)) > f(G), then it’s Regular, for f(F(G)) < f(G) its Singular; and if f(F(G)) = f(G) its unchanged. Table 20 shows the RS analysis of some sample images. The observed R-S differences correlate with the payload size, which validates both the embedding strength and imperceptibility of the proposed steganographic method.

**Table 20:** RS analysis results for some sample images.

Image	R	S	R-S	Estimated payload
Eye.png	5021	4989	32	2%–3%
Tulip.png	6234	6101	133	5%–6%
Monalisa.png	5120	5080	40	3%–4%
Taiwan.png	6010	5900	110	4%–5%

The detect LSB-based embedding RS is a widely adopted steganalysis technique, and the results in Table 20 demonstrate that the proposed approach exhibits very strong resistance to statistical detection. Additional techniques of steganalysis like Chi-square test, robustness against noise attacks and compression, and Sample Pair Analysis (SPA) will be explored in future work.

### Time complexity

The time complexity is denoted in asymptotic Bio-O notation, which shows the growth behavior but does not correspond to an exact numerical value. The time complexity for the Embedding process is as follows: For the chaotic method, it is *O*(*P*), for quantization (with selected chaotic values) it is: Hence, for the worst case, the total time complexity is

$$\begin{aligned} \text {Time} =\;&O(1)\; \text {(Key generation)} \\&+ O(P)\; \text {(Encryption)} \\&+ O(P)\; \text {(Chaotic method)} \\&+ O(P \log P)\; \text {(Quantization)} \\&+ O(P)\; \text {(Embedding)} \end{aligned}$$

Time complexity = $$O(P \log P)$$

Example of chaotic quantization embedding: Monalisa.png (230 $\times$ 216)

Pixels (*P*) = $$W \times H \times C = 230 \times 216 \times 3 = 149040$$

The dominant term in the time complexity is $O(P \log P)$, this term shows that the computational cost increases proportionally as the number of pixels increases with a logarithmic factor. As the base of the logarithm does not affect the complexity, it is omitted.

The logarithmic factor in the time complexity is from the quantization process, which has sorting or ordering operations on pixel values, which results in a complexity of $O(P \log P)$. Since each block requires constant-time chaotic mapping and quantization, the overall process scales linearly with input size. For generating chaotic values and sorting them before assignment, the time complexity is $O(P \log P)$.

### Comparison with recent works

Current research predominantly focuses on either selective protection or encryption mechanisms. In contrast, the proposed framework in ABECIQ combines key generation, its encryption and embedding steganographically into a single system, thereby achieving both high imperceptibility and security. There is no direct quantitative comparison with recent methods due to the differences in objectives and implementation settings and therefore is infeasible. Hence, a qualitative comparison is shown in Table 21 to compare the recent works with the proposed algorithm. From the table, it is observed that the ABECIQ algorithm achieves a high imperceptibility and therefore provides a single unified security framework, thereby distinguishing it from the state-of-the art methods that focus only on specific aspects of security.

**Table 21:** Comparison with recent state-of-the-art methods.

Method	Year	PSNR	Ciphertext randomness	Key sensitivity	Steganalysis resistance	Overhead
Chaotic-LSB	2014	Moderate	Low	Low	Low	Low
AES-RDH	2018	Moderate	Moderate	Moderate	Moderate	Moderate
Cross-image Permutation & Diffusion	2025	High	High	High	Moderate	High
Semantic Selective Encryption	2026	High	High	Moderate	Moderate	Moderate
ABECIQ (Proposed)	2026	Very High	High	High	High (RS)	Moderate

## Conclusion

The proposed study provides a hybrid cryptography-steganography unified framework ABECIQ that combines the genetic algorithm-based key generation, modified Blowfish encryption along with chaotic quantization-based embedding. The security of the proposed method is primarily determined by its key space. With a key size of 128 bits, the total key space is $2^{128}$, making brute-force attacks computationally infeasible under current technological constraints.The method improves the encryption strength, increases key randomness and also achieves high imperceptibility in steganographic embedding. The experimental results show that the ABECIQ also achieves higher values of PSNR and SSIM which indicates minimal distortion along with enhanced performance in terms of execution time. The reliability of the results is confirmed by the statistical analysis which includes mean ± standard deviation and t-test whereas RS analysis shows resistance of the system to steganalysis. However, the proposed work is limited to specific steganalysis approaches like Chi-squate, SPA and to certain evaluation sceanarios in additional to test validating the system’s robustness against compression and noise attacks that need to be explored. Limitations include the need to evaluate compatibility with alternative steganographic schemes such as LSB Matching Revisited (LSBMR), which requires adjustments to align with the embedding and retrieval framework. Future work will focus on extending comparisons with LSBMR and the security analysis along with evaluating the method under various attack conditions which will validate the system’s effectiveness. Furthermore, due to the differences in the evaluation frameworks comparison with deep learning-based approaches is left for future work.

## Data Availability

The datasets used and/or analysed during the current study available from the corresponding author on reasonable request.
